# Conditional quorum-sensing induction of a cyanide-insensitive terminal oxidase stabilizes cooperating populations of *Pseudomonas aeruginosa*

**DOI:** 10.1038/s41467-019-13013-8

**Published:** 2019-11-01

**Authors:** Huicong Yan, Kyle L. Asfahl, Na Li, Feng Sun, Junwei Xiao, Dongsheng Shen, Ajai A. Dandekar, Meizhen Wang

**Affiliations:** 1 School of Environmental Science and Engineering, Zhejiang Gongshang University, Hangzhou, China; 2 Zhejiang Provincial Key Laboratory of Solid Waste Treatment and Recycling, Hangzhou, China; 3 Department of Medicine, University of Washington, Seattle, WA USA; 4 Department of Microbiology, University of Washington, Seattle, WA USA

**Keywords:** Social evolution, Bacteriology, Bacterial genetics

## Abstract

*Pseudomonas aeruginosa*, an opportunistic pathogen of humans, uses quorum sensing (QS) to regulate the production of extracellular products that can benefit all members of the population. *P. aeruginosa* can police QS-deficient cheaters by producing hydrogen cyanide, which is also QS regulated; however, the mechanism by which cooperators selectively protect themselves from the toxicity of cyanide remained unresolved. Here, we show that a cyanide-insensitive terminal oxidase encoded by *cioAB* provides resistance to cyanide, but only in QS-proficient strains. QS-deficient cheaters do not activate *cioAB* transcription. QS-mediated regulation of *cioAB* expression depends on production of both cyanide by cooperators (which is QS regulated) and reactive oxygen species (ROS) from cheaters (which is not QS regulated). This type of regulatory system allows cooperating populations to respond, via ROS, to the presence of cheaters, and might allow them to defer the substantial metabolic cost of policing until cheaters are present in the population.

## Abstract (web summary)

Quorum sensing (QS) regulates production of ‘public goods’ by *Pseudomonas aeruginosa*, which releases toxic hydrogen cyanide to constrain QS-deficient cheaters. Here, Yan et al. show that QS-proficient strains protect themselves by producing a cyanide-insensitive enzyme in response to reactive oxygen species released by cheaters.

## Introduction

The opportunistic pathogen *Pseudomonas aeruginosa* uses a complex QS circuit to regulate the production of numerous shared or public goods, including secreted proteases and other virulence factors^1,2^. The QS circuitry in *P. aeruginosa* consists of two acyl-homoserine lactone (AHL) signal-receptor pairs. In the *las* system, the signal synthase LasI produces a signal, *N*-3-oxo-dodecanoyl homoserine lactone (3OC12-HSL), which binds to the transcription factor LasR. In the second, the *rhl* system, the signal synthase RhlI produces *N*-butyryl-homoserine lactone (C4-HSL), which is bound by the transcription factor RhlR. The LasI-R system regulates the RhlI-R system, so null mutations in LasR have the effect of inactivating both QS systems^1,2^. When *P. aeruginosa* is passaged in conditions where QS is required to obtain carbon and energy for growth, such as in media containing the protein casein as the sole carbon and energy source, social cheaters reproducibly emerge in the population^3,4^. These social cheaters invariably have LasR-inactivating mutations^3,4^.

In microbial communities, the most beneficial strategies for individual cells can be at odds with the best strategy for the entire population. Cooperating individuals may produce a costly public good that can be shared and serve to enhance fitness of the entire population. However, selection often favors cheating individuals that avail themselves of public goods while avoiding investment, ultimately risking cooperative collapse^5,6^. Several mechanisms have been described to stabilize cooperation in populations, including delayed production of public goods^7^, and independent regulation of multiple public goods^8^.

We have shown that *P. aeruginosa* cooperators secrete hydrogen cyanide (HCN) as a means to “police” cheaters^9^. Policing depends on activation of the RhlI-R QS circuit^9^, which regulates the genes that code for production of the hydrogen cyanide synthase^10^. Deletion of either RhlR or the genes that encode the cyanide synthase results in a loss of policing by cooperators. In policing, pleiotropic regulation of both toxin and immunity mechanisms allows a cooperating population to punish neighboring cells that lack sufficient relatedness at a specific locus^11^. Without this policing mechanism, the cheaters outcompete cooperators and cooperating populations collapse, as there is ultimately an insufficient number of cooperators to continue growing in conditions where QS is required for growth, a type of “tragedy of the commons”^9^.

Policing comes at a cost to the cooperators: in pure cultures, bacteria that produce cyanide grow more slowly and to a lower yield than bacteria that do not make cyanide^9^. It is not apparent from previous studies of *P. aeruginosa* QS how cooperators might protect themselves against cyanide intoxication. The *P. aeruginosa* genome contains a conserved cyanide-insensitive terminal oxidase that, unlike hydrogen cyanide synthase, has not been shown to be upregulated in published QS transcriptomes^12,13^. We are interested in the mechanism of self-protection against cyanide intoxication by cooperating populations of *P. aeruginosa*.

Here, we show that QS-proficient bacteria are more resistant to death in the presence of cyanide than QS-deficient cheaters. We further demonstrate this difference in survival reflects conditional quorum regulation of genes encoding a cyanide-insensitive terminal oxidase, *cioAB*.

## Results

### Cheaters are more sensitive to cyanide than cooperators

**Fig. 1:**
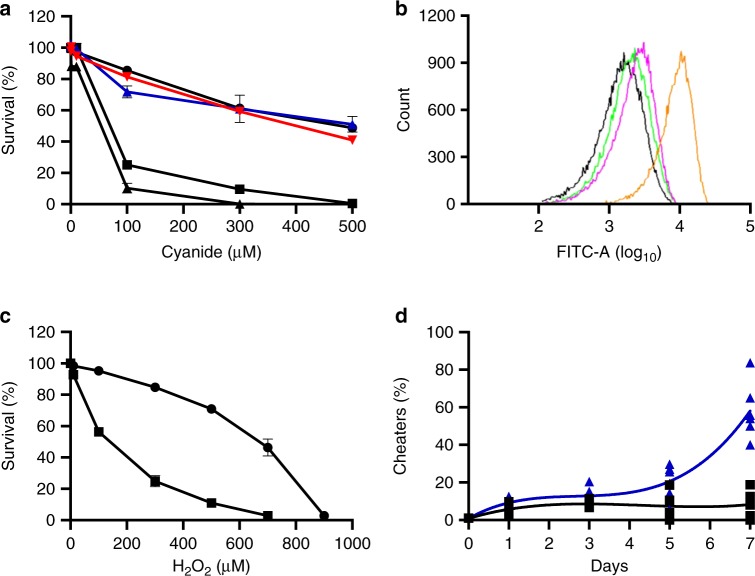
Wild-type PAO1 is more resistant to cyanide and ROS intoxication than a LasR mutant. **a** Survival of WT PAO1, LasR-mutant, RhdA-mutant, PA4133-mutant, or CioA-mutant, grown in the presence of different concentrations of cyanide with initial OD_600_ of 0.005. The cell density was measured after 16 h of growth. Black circles, WT PAO1; red inverted triangles, RhdA-mutant; blue triangles, PA4133-mutant; black squares, LasR-mutant; black triangles, CioA-mutant. **b** ROS production from the WT PAO1 and LasR-mutant when grown in LB-MOPS with or without 150 μM CN^−^ for 40 min. ROS generation was measured by FTC fluorescence. Black, WT PAO1 without cyanide; green, WT PAO1 with cyanide; pink, LasR-mutant without cyanide; orange, LasR-mutant with cyanide. **c** Survival of WT PAO1 or LasR-mutants in the presence of hydrogen peroxide after 16 h of growth. Circles, WT PAO1; squares, LasR-mutants. **d** Cheater frequency in the presence or absence of catalase. The initial frequency of cheaters was 0.01. Black, competition without catalase; blue, competition with 25 U/mL of catalase added daily. All experiments were performed with a minimum of three biological replicates. Lines represent best-fit polynomial regressions for each condition. For **a**, **c** means, s.e.m., and individual biological replicates are shown and in some cases error bars are too small to be seen

Initially, we asked if cooperators could better survive cyanide intoxication than cheaters, by comparing the resistance either of the cooperators or the cheaters to exogenously added cyanide. We grew wild-type (WT) *P. aeruginosa* PAO1, or a LasR-null mutant of PAO1, in LB-MOPS broth with a range of added potassium cyanide (0–500 µM), which mimics the maximal concentration of cyanide ion (CN^−^) produced by PAO1 in stationary phase^14^. After 16 h, survival of WT PAO1 under 100 µM of CN^−^ stress was 3.5-fold higher than for LasR-null mutants (Fig. 1a); the median lethal concentration (LC_50_) of CN^−^ was ~470 µM for the WT and 60 µM for LasR mutants, respectively (Fig. 1a).

Hydrogen cyanide inhibits cytochrome C oxidase in susceptible cells, resulting in production of reactive oxygen species (ROS)^15,16^. Therefore, we reasoned that ROS production should be greater from a population of LasR-null mutants than the WT, when grown in the presence of cyanide. To measure the generation of ROS, we used a fluorescence assay as described in “Materials and Methods”. ROS production from a population of LasR-mutants in the presence of 150 μM CN^−^ was 2.2-fold greater than in the absence of CN^−^ (Fig. 1b). WT populations showed very little increase in ROS production when exposed to CN^−^. Resistance to another generator of reactive oxygen species, H_2_O_2_, was also higher in WT PAO1 than in LasR-mutants (Fig. 1c).

We next asked if the outcome of competition between WT PAO1 and LasR-null mutants would be affected by the addition of catalase, as addition of catalase can protect against ROS damage by converting peroxide to water and oxygen. When the WT and LasR-mutants are passaged in casein broth, the LasR-mutants and WT come to an equilibrium of ~20% cheaters^3,4,9^ (Fig. 1d). However, when 200 units catalase were added to the culture daily with passage, cooperators lost the ability to police cheaters, which came to a high percentage in the population (Fig. 1d). At this point, the population could no longer be propagated for lack of sufficient numbers of cooperators.

### QS activation alone is insufficient to confer cyanide resistance

A survival difference between the WT and LasR-mutants in the presence of cyanide, along with evidence for the regulation of cyanide production by RhlR^9^, implies that the mechanism of cyanide resistance is QS-regulated. Three mechanisms, each involving different gene products, have been reported to ameliorate cyanide toxicity in *P. aeruginosa*^17–19^. However, none of these gene products are known to be QS-regulated^12,13^. One of the gene products, the enzyme Cio, a cyanide insensitive terminal oxidase encoded by *cioAB*, allows *P. aeruginosa* to respire oxygen in the presence of cyanide. Complementing the function of Cio are the gene products of a six-gene cluster, PA4129-4134, that synergizes with Cio through an unknown mechanism to protect against cyanide intoxication. Finally, the protein RhdA, a cyanide sulfur-transferase, can convert cyanide to thiocyanate, which is less toxic.

**Fig. 2:**
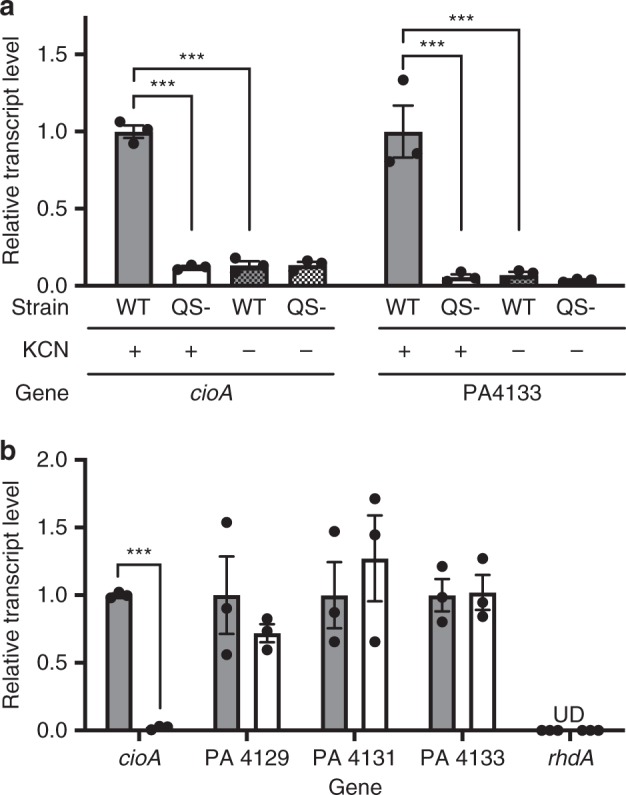
Regulation of *cioA* (PA3930) by QS is conditional on the presence of cyanide. **a** Expression of *cioA* (PA3930) and PA4133 in the WT PAO1 (black, “WT”) and LasR-RhlR-double mutant (white, “QS-”). Solid, in the presence of 150 μM KCN; grid, in the absence of 150 μM KCN. **b** *cioA*, PA4129, PA4131, PA4133, and *rhdA* expression from either WT PAO1 (“policing”, black) or RhlR-null cooperators (“non-policing”, white) when grown in co-culture with cheaters, with the populations separated by dialysis membranes. Expression was measured by RT-PCR with *proC* used as reference gene. Means, s.e.m., and individual biological replicates are shown (*n* = 3). Statistical significance determined by unpaired Student’s *t*-test; ***p < 0.001. UD, undetected

Previous reports about QS-regulated genes in *P. aeruginosa* have not identified the *cioAB*, *rhdA* or PA4129-4134 genes as RhlR- or LasR-regulated^12,13^. A possible explanation for this paradox would be that regulation of one or all depends on the presence of cyanide. To determine which of these gene products might be contributing to the cyanide resistance phenotype, we measured transcript levels of *cioA* (PA3930), *rhdA* (PA4956), and PA4133 (in the six gene cluster) in WT PAO1 in the presence and absence of 150 µM potassium cyanide. In the presence of cyanide, the expression levels of *cioA* and PA4133 were about 7-fold and 14-fold higher in WT PAO1 than in the absence of cyanide, suggesting a possible role for both gene products in protection against cyanide intoxication (Fig. 2a). Expression levels of *rhdA* were too low to be detected in any of the conditions tested. These results were consistent with the idea that the presence of cyanide was a condition required for the expression of resistance genes.

We next asked if QS was also required for induction of either *cioA* or PA4133. We measured transcript levels of both of these genes in WT PAO1 as compared with LasR-RhlR-double mutants, and found that the QS-proficient WT strain had eightfold more *cioA* transcript and 17-fold more PA4133 transcript than the QS-deficient strain (Fig. 2a), in the presence of cyanide. Together with the differential susceptibility of each of these strains to cyanide (Fig. 1a), the results suggested to us that self-protection against cyanide policing is in part conditional on the policing mechanism itself or the ecological consequences of policing within a mixed population.

One alternative explanation for these results is that Cio expression is dependent on the intracellular production of cyanide, not QS activation *per se*. That is, because cyanide is produced at very low levels in LasR-null mutants^20,21^, it is possible that the protection conferred by Cio is not activated in these strains as those cells do not make cyanide. To determine if endogenously-produced cyanide could activate Cio (or other protective mechanisms) in the LasR-null mutant, we introduced an additional copy of the *hcnABC* operon at neutral site on the chromosome of both WT and LasR-null mutants, with the genes under control of the arabinose-inducible P*araBAD* promoter. Addition of arabinose to cells harboring this construct results in QS-independent cyanide production. We competed these strains against each other in casein broth supplemented with L-arabinose. The LasR mutant fared more poorly in competition when arabinose was added (Supplementary Fig. 1), which indicates that endogenous cyanide production in a LasR mutant does not activate the cyanide protection mechanism.

### *cioAB* is upregulated in cooperators when cheaters are present

We reasoned a second potential explanation for the conditional regulation of self-protection to cyanide could be response to the culture conditions where the population is actively policed. To test this idea, we measured gene expression in the context of co-cultures between two different types cooperators and cheaters. In one configuration, WT PAO1 should police LasR-mutants through the production of cyanide (Fig. 1d) (a “policing” condition)^3,4,9^. In another, a RhlR-null cooperator cannot police a RhlR-LasR-double mutant cheater (a “non-policing” condition)^9^. We separated cheaters and cooperators in these experiments with dialysis membranes and compared expression by cooperators of *cioA*, PA4129, PA4131, PA4133, or *rhdA* in both the “policing” and “non-policing” conditions. We added analysis of PA4129 and PA4131 in this analysis because differences in PA4133 expression were markedly different than that seen in the presence/absence of cyanide (Fig. 2a). There were no significant differences in expression of PA4129, PA4131, PA4133, or *rhdA* between the policing and non-policing conditions (Fig. 2b). However, expression of *cioA* in the policing condition was nearly 50 times the level observed in the non-policing condition. Together, these data are consistent with the hypothesis that *cioAB* expression is the relevant factor in QS-mediated protection against self-intoxication, and that QS regulation of these genes depends on the QS-regulated production of cyanide in the conditions of our experiments. This is a type of AND logic gate: gene regulation depends on a QS transcription factor and also an environmental condition or cue^22^, and in this case the cue is likely environmental ROS.

**Fig. 3:**
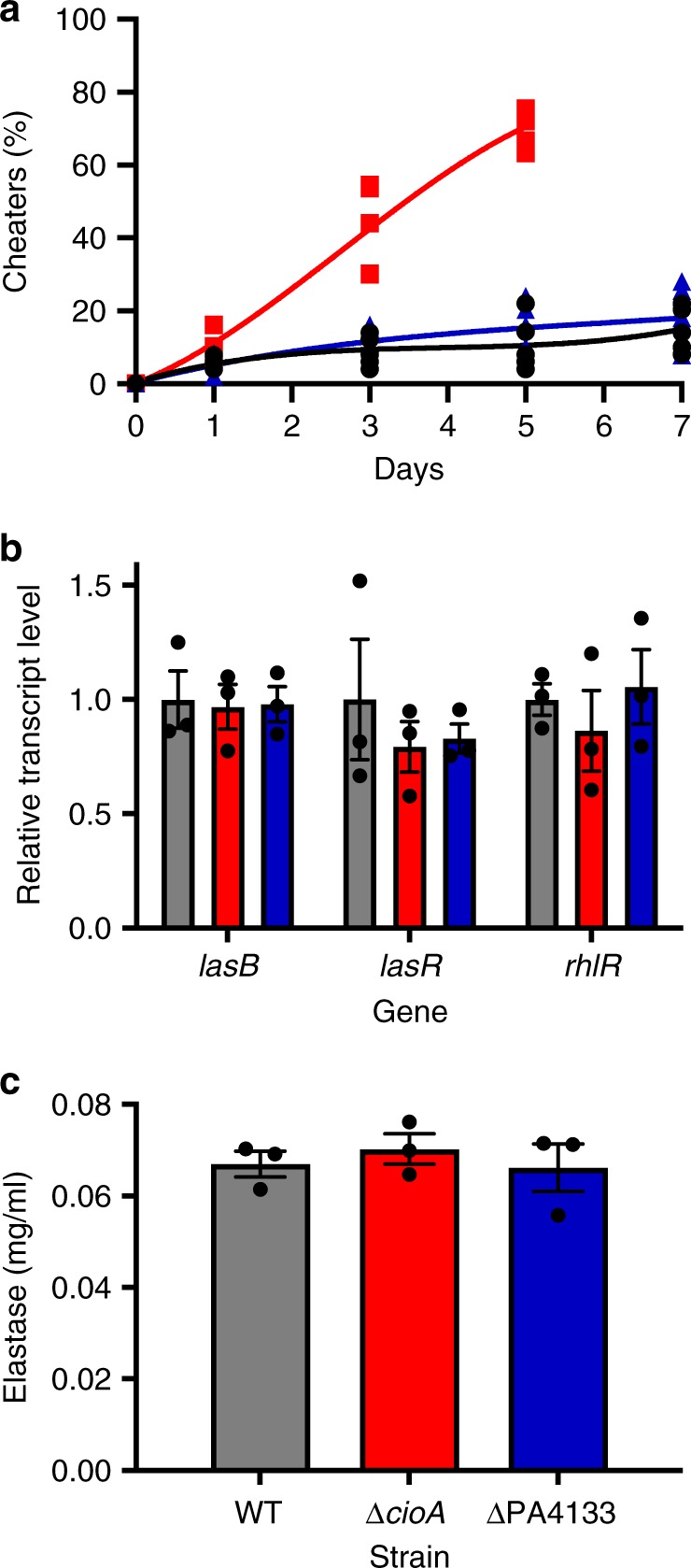
The cyanide insensitive terminal oxidase Cio mediates self-protection of cooperators. **a** Competition between WT PAO1 (black), CioA-null cooperator (red), or PA4133-null cooperator (blue) and LasR-null mutants. The initial frequency of cheaters was 0.01. Five biological replicates were performed. Lines represent best-fit polynomial regressions for each condition. **b** Expression of *lasB, lasR* and *rhlR* in WT PAO1 (black), CioA-null mutant (red), and PA4133-null mutant (blue). **c** Elastase production by WT PAO1 (black), CioA-null mutant (red), and PA4133-null mutant (blue). For **b**, **c** means, s.e.m., and individual biological replicates are shown (*n* = 3)

Our earlier observation that a CioA-null mutant was more sensitive to cyanide intoxication than WT PAO1 (Fig. 1a) is consistent with the idea that Cio plays an important role in policing. To ascertain if the CioA-mutants lost the ability to police cheaters, by virtue of no longer having an advantage over LasR-mutants in terms of cyanide tolerance, we grew CioA-mutants in co-culture with LasR-mutants at a starting ratio of 99:1. CioA-mutants are known not to have a defect in cyanide production^17^. Within 3 days, the cheater proportion increased to about 50% (Fig. 3a). By contrast, both WT PAO1 and the PA4133-null mutants were able to police cheaters and the frequency plateaued at 20%, as previously described^3,4,9^. Although the CioA-null co-culture could not be propagated past day 5 for lack of sufficient cooperators, there was no difference in population productivity of QS-regulated exoproducts compared with WT PAO1, as measured by total elastase and cyanide production by CioA-null or PA4133-null cooperators (Supplementary Fig. 2). None of these deletions adversely affect the growth rate or final cell density in either LB-MOPS or casein broth (Supplementary Fig. 3). We also did not observe differences in either the expression of *lasB*, *lasR*, or *rhlR* (Fig. 3b) or the production of public good elastase (Fig. 3c) between these three strains. These results indicated that Cio, not the gene products of PA4129-4134, is the critical element of QS-regulated self-protection from cyanide intoxication in the conditions of our experiments.

### Interference with CioA prevents policing

Because a CioA-null cooperator is susceptible to a tragedy of the commons (Fig. 3a), we reasoned that interfering with the expression of Cio in strain PAO1 could increase the frequency of QS-deficient cheaters, resulting in a failure to propagate. To do so, we added copper (in the form of copper sulfate) to the growth medium, which has been reported to decrease *cioAB* expression while also stabilizing the non-Cio high-affinity aerobic terminal oxidases^23^. The predicted effect of copper sulfate addition is twofold: cooperators would have less Cio, and stabilization of terminal oxidases that would otherwise be poisoned by cyanide might protect cheaters from policing.

**Fig. 4:**
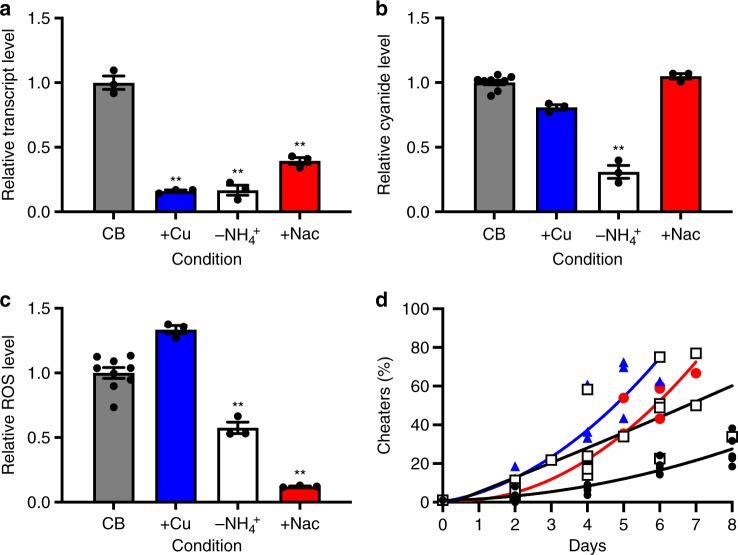
Inhibition of *cioA* expression as a potential strategy to destabilize populations of *P. aeruginosa*. Expression of **a** *cioA* and **b** production of cyanide when WT PAO1 is grown in casein broth (CB, black) with the addition of 1 mM copper sulfate (+Cu, blue). Shown in comparison are casein broth without ammonium sulfate (−NH_4_^+^, white), and media with added *N*-acetylcysteine (+Nac, red). **c** ROS levels in the same conditions. **d** Cheater frequency when WT cooperators and LasR mutants are grown in these conditions. In all co-cultures, the starting ratio of WT:LasR mutants was 99:1. At least three biological replicates were performed. Lines represent best-fit polynomial regressions for each condition. For **a**–**c**, means, s.e.m., and individual biological replicates are shown. Statistical significance determined by paired Student’s *t*-test versus CB alone; **p < 0.01. UD; undetected

We first tested the effect of copper sulfate addition to casein broth in monocultures of WT PAO1 and found a reduction in expression of *cioA* nearly as much as if ammonia, a necessary substrate for hydrogen cyanide synthesis^24^, was not present (Fig. 4a). This finding was not paralleled by a decrease in cyanide synthesis, where absence of ammonium sulfate decreases cyanide production significantly compared with the control (Fig. 4b)^24^. These data show that addition of copper sulfate decreased *cioA* expression in cooperators but had little or no effect on cyanide production. Addition of 1 mM *N*-acetylcysteine, an ROS scavenger, did not reduce *cioA* expression to the same degree as copper sulfate and, similar to copper sulfate addition, did not affect cyanide production (Fig. 4a, b).

We then measured ROS generation in cultures of WT PAO1 treated with copper sulfate, *N*-acteylcysteine, or grown in the absence of ammonium sulfate. ROS production was significantly reduced in both ammonium-sulfate-free media or when *N*-acetylcysteine was added (Fig. 4c). Addition of copper sulfate, which reduced *cioA* expression, had an insignificant impact on hydrogen cyanide production and therefore ROS generation (Fig. 4b, c).

Finally, we asked if LasR-mutant cheaters could increase to an unsustainable frequency in copper-sulfate treated populations, reasoning that interference with *cioA* expression may produce a similar effect as observed in competitions with a CioA-null mutant (Fig. 3a). We grew WT PAO1 and LasR-mutants together in casein broth at a starting ratio of 99:1. Again, because of cyanide policing, the LasR mutants come to an equilibrium with the wild-type PAO1 at a frequency of 20–40% (Fig. 4d). Consistent with our hypothesis, the addition of copper sulfate resulted in a dramatic increase in the frequency of LasR-mutant cheaters, ultimately exceeding 60% with a concomitant tragedy of the commons where the population could no longer be propagated. We observed a similar consequence when *N*-acetylcysteine was added to or ammonium sulfate was absent from the growth medium, consistent with prior reports^24^.

## Discussion

Maintenance of cooperation in microbial populations is an evolutionary conundrum for which several possible solutions have been described. In one of these, metabolic policing, quorum-sensing cooperators secrete an intoxicant to punish cheaters that do not produce public goods^9^. In *P. aeruginosa*, one such intoxicant is cyanide. Because cyanide production is QS-regulated, a reasonable assumption would be that QS regulates both the production of cyanide and the necessary enzymes for detoxification. However, previously published studies of QS-regulated genes have not identified candidates for this function. In the present work, we demonstrated that QS cooperators are more resistant to cyanide intoxication than QS-null LasR mutants, which are cheaters under the conditions of our experiments (Fig. 1a). The WT is also more resistant to damage from the generation of ROS than the LasR mutant (Fig. 1b).

We demonstrated that the regulation of genes encoding the cyanide-insensitive terminal oxidase Cio is involved in protection of cooperators from cyanide intoxication, and that *cioAB* is co-induced by QS and cyanide (Fig. 2a). This feature of QS gene expression by AND gates is characteristic of *P. aeruginosa*^25^, and explains why *cioAB* were not identified as QS regulated in prior transcriptomic studies^12,13^.

While expression of genes in the *hcnABC* operon have been found to be robustly QS-controlled in multiple growth media and at multiple stages of growth^12,13,26^, translation and resulting elaboration of measurable cyanide by QS-proficient strains is delayed until cells are firmly in stationary phase^27^, further stalling cyanide-dependent induction of *cioAB* and preventing discovery of these genes as QS-dependent. The mechanism by which cyanide concentrations in the environment or the resulting ecological effects are sensed and integrated into the QS-dependent transcriptional program of *P. aeruginosa* remains elusive.

Other candidates for QS-regulated cyanide detoxification, including the gene products of PA4129-4134 and *rhdA*, were not involved under the conditions of our experiments and *rhdA* was not expressed (Fig. 2b). However, our data do not exclude a role for the products of these genes in *P. aeruginosa* cyanide detoxification; they are merely not the key players in QS-mediated resistance to policing. In particular, we show that PA4133 is, like *cioAB*, conditionally regulated by QS when cyanide is present. There may be other circumstances where cyanide production is important, including in interspecific competition^28^, where the contribution of these other factors may be comparatively greater.

We also showed that supplementation of the growth media with copper sulfate, which alters the efficiency of non-Cio cytochrome oxidases^29^, or *N*-acetylcysteine, an ROS scavenger, has the potential to induce the collapse of a mixed population of *P. aeruginosa*. We suspect that they do so by reducing the difference in cyanide susceptibility between the WT and LasR mutants, pointing to a potential use for this strategy in settings where a population collapse of *P. aeruginosa* would be desirable. When cooperators are no longer able to selectively intoxicate cheaters effectively, in addition to the metabolic cost of cooperative protease secretion, they suffer the metabolic cost of policing without a commensurate benefit, and cheaters rise to an unsustainable frequency (Fig. 4d).

In a setting where policing is the major restraint on cheaters, there would be a selective pressure for cheaters to develop a non-QS-regulated mechanism to resist cyanide toxicity. This might take the form of constitutive expression of *cioAB*, upregulation of one of the other genes involved in cyanide detoxification, or by some other mechanism. However, even in 30-day passages^3,4^ such variants did not appear to emerge. It may be that the cost to the cheater of this kind of a regulatory rewiring would not exceed the benefit, as the selective pressure (at least in the conditions of our experiments) is not very strong: even with policing, cheaters still come to comprise ~20% of the population.

Our work illustrates the intricacy of QS regulation in *P. aeruginosa* and the conditional nature of some elements of the regulon. This kind of regulation might allow bacterial populations to behave differently depending on environmental cues including, in this case, the presence of cheaters. For applications where a loss of quorum sensing induction in the population may be beneficial, such as in acute infections or biofouling^30–32^, our work points to new avenues of development for biological control of *P. aeruginosa*.

## Methods

### Bacterial strains and growth conditions

Bacterial strains used in this study are listed in Supplementary Table 1. Bacteria were grown in minimal medium with 1% (w/v) sodium caseinate as the sole carbon source (“casein broth”)^4^ or in LB buffered with 20 mM *N*-morpholinopropanesulfonic acid (LB-MOPS)^33^. Unless otherwise specified, cultures were grown in 16-mm test tubes containing 4 ml media, with shaking (225 RPM) at 37 °C. Cell densities in LB-MOPS were determined as the optical density at 600 nm using a spectrophotometer.

All mutants used in this study were generated using homologous recombination as previously described^34^. PCR-amplified DNA fragments flanking *cioA*, PA4133, and *rhdA* were cloned into pEXG2^34,35^. Briefly, *Escherichia coli* S17-1 containing pEXG2 constructs were mated with PAO1. Transconjugants were selected on Pseudomonas Isolation Agar (PIA) containing gentamicin. Deletion mutants were selected on PIA containing 5% (w/v) sucrose. Mutants were confirmed by PCR of genomic DNA.

The arabinose-inducible *hcnABC* construct was generated using *E. coli*-mediated homologous recombination as previously described^36^. Fragments encompassing the *araC* gene and P*araBAD* promoter, the *hcnABC* operon, and the pUC18T-miniTn7T-GmR backbone were PCR-amplified using primers with homologous ends from pJN105 plasmid DNA, PAO1 genomic DNA, and pUC18T-miniTn7T-GmR plasmid DNA, respectively. The complete plasmid construct was then electroporated, along with helper plasmid pTNS2^37^, into PAO1 and the LasR-null mutant to facilitate insertion at the *att* site in each strain. Transformants were selected on LB agar containing gentamicin and confirmed by PCR.

### Bacterial resistance to CN^−^ or H_2_O_2_

Fresh colonies of *P. aeruginosa* PAO1, LasR-null, PA4133-nul, RhdA-null, or CioA-null strains were inoculated into 4 ml of LB-MOPS. From these initial cultures, 0.4 ml was subcultured into 4 ml fresh LB-MOPS, and grown to mid-log phase (OD_600_ of 0.5). We inoculated 40 μl from these cultures into 4 ml of fresh LB-MOPS (an initial OD_600_ of 0.005) in the presence of 0–500 μM KCN or 0–15 mM H_2_O_2_. We then calculated a survival ratio: the number of cells in the treated groups divided by the number in the untreated group. All experiments were performed in five replicates.

### Competition experiments

We combined two strains at an initial OD_600_ of 0.05 in casein broth. The initial ratio of cooperators (PAO1, CioA-null or PA4133-null) to LasR-null was 99:1, as determined by OD_600_ from overnight growth in LB-MOPS. In some experiments, 25 U/ml catalase was added to casein broth. At 24 h intervals, we transferred 50 μl to fresh casein broth with or without catalase. Periodically, cells were plated for isolated colonies on LB agar. Cheater abundance was determined by patching colonies on skim milk agar, as previously described^4^; we enumerated at least 100 colonies for each condition at each timepoint.

### Measurement of ROS, CN^−^, and elastase

Reactive oxygen species were detected using a 2′,7′-dichlorofluorescein diacetate (DCFH-DA) probe^24^. Briefly, DCFH-DA was added to cultures at a final concentration of 10 μM. After incubation in the dark at 37 °C for 30 min, cells were washed with PBS to thoroughly remove extracellular DCFH-DA. The reaction of DCFH-DA with ROS generates a fluorescent, oxidized derivative, DCF (F). Individual cell fluorescence was measured using a CytoFlex S (Beckman Coutler, USA) flow cytometer in the FITC channel. Events were analyzed using CytExpert 2.0 software (Beckman Coulter). As the experiments were monocultures grown in LB-MOPS, all events were counted (i.e., there was no exclusion gate). Alternatively, total DCF (F) levels were measured in a SpectraMax® i3 plate reader (Molecular Devices, USA) (488 nm excitation, 525 nm detection). The relative ROS level was calculated by dividing the DCF(F) level by that of PBS.

Cyanide concentrations were determined using a CN^−^ ion selective electrode (Cole-Parmer, USA). Briefly, the overnight culture was adjusted to pH 12.0 with NaOH, and then CN^−^ ion detected by conductivity. A standard curve of KCN was made for calculation of the CN^−^ concentrations in samples.

Elastase production was measured using the Pierce Fluorescent Protease Assay kit (Thermo, USA). Briefly, the culture was centrifuged at 15,000 g for 5 min. A total of 100 μl of supernatant was mixed with 100 μl of FTC-Casein working reagent, and incubated for 45–60 min in the dark at room temperature. The fluorescence at 450 nm was detected using a SpectraMax® i3 plate reader (Molecular Devices, USA).

### RT-qPCR

Cells were pelleted by centrifugation and RNA isolation and cDNA synthesis were performed as described elsewhere^38^. One microliter of cDNA was used as the template in quantitative PCR (qPCR) amplification with designed primers (Supplementary Table 2). After 40 cycles (94 °C for 10 min, 94 °C for 5 s, 55 °C for 15 s, 72 °C for 10 s), a melting curve analysis was performed (60–95 °C) to verify the specificity of amplicons. The relative level of expression (*Q*) for a given gene was calculated based on the formula *Q* = 2^−ΔCt^. Each Ct value represents the average of three replicates, where ΔCt represents the target gene Ct minus the Ct of the housekeeping gene *proC*^39^.

### Reporting summary

Further information on research design is available in the Nature Research Reporting Summary linked to this article.

## Supplementary information

Supplementary Information

Reporting Summary

Source Data

## Data Availability

The datasets generated and analysed during the current study are available in the accompanying Source Data file. The strains used in these studies are available from the corresponding authors on reasonable request.
