# Supplementary material for: Conditional quorum-sensing induction of a cyanide-insensitive terminal oxidase stabilizes cooperating populations of Pseudomonas aeruginosa
Source: Nat Commun. 2019 Nov 1;10:4999. doi: 10.1038/s41467-019-13013-8 (PMC6825135; doi:10.1038/s41467-019-13013-8)
Supplement: Supplementary file 1 — Supplementary Information [file 41467_2019_13013_MOESM1_ESM.pdf]

Supplementary information for

Conditional quorum-sensing induction of a cyanide-insensitive terminal oxidase stabilizes cooperating populations of *Pseudomonas aeruginosa*

Yan et al. (2019)

Supplementary Table 1. Bacterial strains and plasmids used in this study.

Strains or plasmids	Genotype and/or relevant characteristics	Reference or source
P. aeruginosa		
PAO1-UW	Wild-type	1
PAO1 Δ*lasR*	*lasR* null mutant	2
PAO1 Δ*rhlR*	*rhlR* null mutant	2
PAO1 Δ*lasRrhlR*	*lasR rhlR* null double mutant	2
PAO1 Δ*hcnC*	*hcnC* null mutant	2
PAO1 Δ*cioA*	*cioA* null mutant	This study
PAO1 Δ*rhdA*	*rhdA* null mutant	This study
PAO1 ΔPA4133	PA4133 null mutant	This study
PAO1 ara-hcn1	Wild-type UW strain with L-arabinose-inducible *hcnABC* inserted at the *att* site	This study
PAO1 Δ*lasR* ara-hcn1	*lasR* null mutant with L-arabinose-inducible *hcnABC* inserted at the *att* site	This study
E. coil		
S17.1 (λ *pir*+)	Conjugal transfer strain for pEXG2 plasmids	
DH5α	Host strain for pUC18T plasmid recombination	
Plasmids		
pEXG2	Gm^r*mob*, *sacB*, *lacZα*	3
pEXG2-Δ*cioA*	pEXG2 derivative carrying the *cioA* flanking regions	This study
pEXG2-Δ*rhdA*	pEXG2 derivative carrying the *rhdA* flanking regions	This study
pEXG2-ΔPA4133	pEXG2 derivative carrying the PA4133 flanking regions	This study
pUC18T-mini-Tn7T-P*araBAD*-1*hcnABC*-Gm	pUC18 derivative carrying mini-Tn7T transposable cassette containing *araC* and the *araBAD* promoter immediately upstream of the 2995 bp *hcnABC* operon	This study
pTNS2	Helper plasmid for transposition of mini-Tn7T cassette	4

Supplementary Table 2. RT-PCR primers used in this study.

Target gene	Primer name	Sequence (5’-3’)	*Amplicon (bp)*	T_m (℃)
lasR	*lasR*-F	cagaagatggcgagcgaccttg	102	59.9
	lasR-R	cgggtagttgccgacgatgaag		59.7
rhlR	*rhlR*-F	gcgaccagcagaacatctccag	122	59.6
	rhlR-R	cgggttggacatcagcatcgg		59.6
lasB	*lasR*-F	cagaagatggcgagcgaccttg	102	62
	lasR-R	cgggtagttgccgacgatgaag		61
PA4129	PA4129-F	gaggacaagaacgtcgagga	102	57
	PA4129-R	ctagcgtcggcggaacaa		58
PA4131	PA4131-F	tacattttcggcgcgacctt	125	59
	PA4131-R	caggtatagccgcaccagac		58
PA4133	PA4133-F	aactggttcttcggctcgtt	165	58
	PA4133-R	ggtcaggaagaagcctacgg		58
cioA	*cioA*-F	atcttcaacccgtcgttccc	143	58
	cioA-R	catcgagagcatcttgcgga		58
cioB	*cioB*-F	ctggtgaccttctatgcgct	130	58
	cioB-R	ggatgatgttcggccacagg		59
rhdA	*rhdA*-F	ctaccactatctcaacggcgg	176	58
	rhdA-R	catcccataccgccaggtc		58
proC	*proC*-F	cgtcgtggtcctgtcggtca	100	60
	proC-R	ggcggcgatggagacgatca		60

Supplementary Figure 1

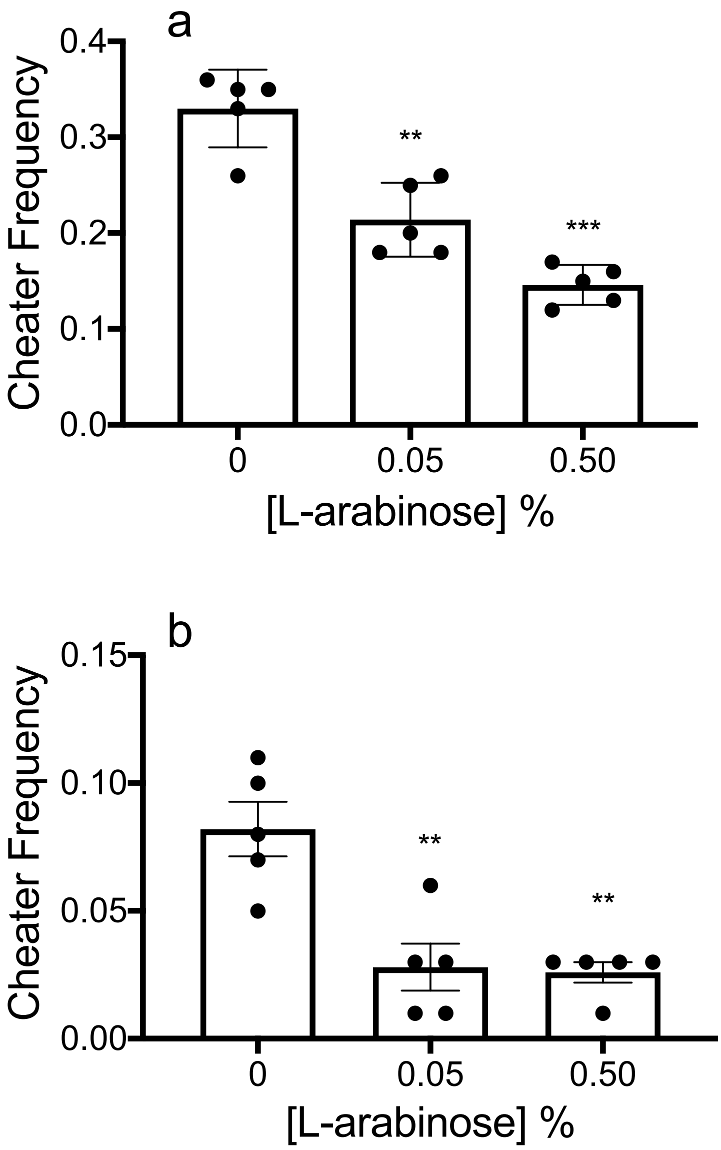

Supplementary Figure 1. Endogenous production of HCN negatively affects LasR mutant enrichment in casein broth competitions with WT. WT and LasR mutant strains harboring an L-arabinose-inducible copy of *hcnABC* were competed for 24 h in casein medium from a combined starting OD_{600}=0.05 and target cheater frequency of 0.1 **(a)** or 0.01 **(b)**. All other experimental conditions are the same as described for other competitions in Materials and Methods of the main text. Statistical significance determined by unpaired Student's *t*-test vs 0% L-arabinose alone (n=5); ***, $p < 0.001$; **, $p < 0.01$. Means, s.e.m., and individual biological replicates are shown.

Supplementary Figure 2

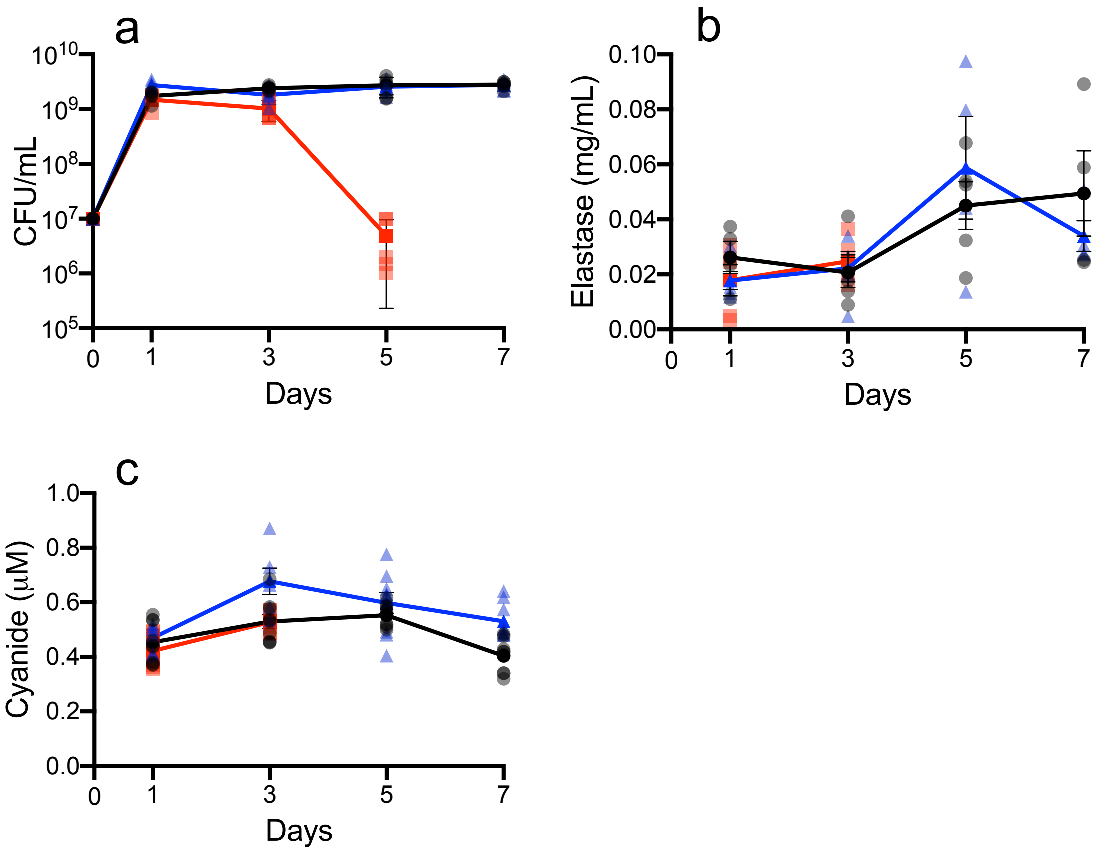

Supplementary Figure 2. Co-culture productivity and QS-regulated cooperative behaviors. In co-culture competitions between LasR mutant cheaters and WT cooperators (black circles), PA4133-null cooperators (blue triangles), or *cioA*-null cooperators (red squares), productivity was measured as CFU/mL **(a)** at each indicated timepoint. QS-regulated cooperative behaviors were also measured at these timepoints as elastase production **(b)** or cyanide concentration **(c)**. See Materials and Methods for experimental procedures. A minimum of five biological replicates were performed and means, s.e.m., and individual replicates are shown.

Supplementary Figure 3

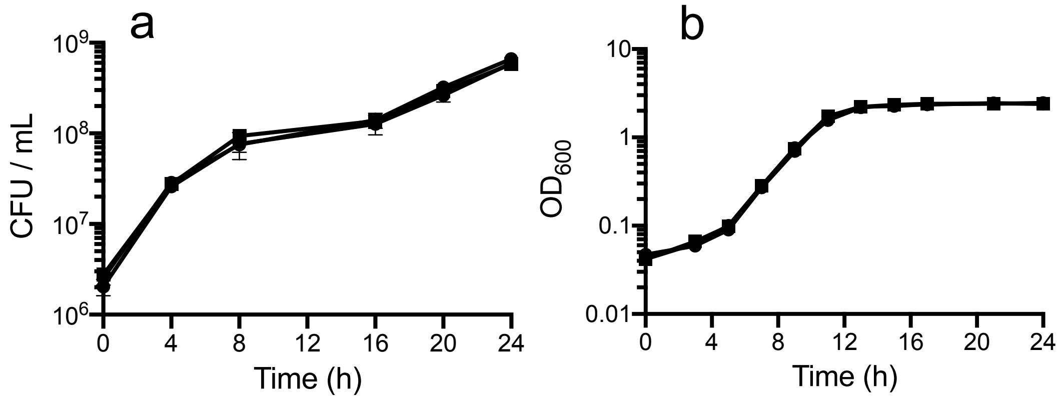

Supplementary Figure 3. Growth rates of WT PAO1 (circle), a CioA-mutant (square), and a PA4133-mutant (triangle). Bacteria were inoculated from overnight LB-MOPS cultures into casein broth **(a)** or LB-MOPS **(b)**. Cell density in casein broth was measured by colony counts and in LB-MOPS by optical density at 600 nm. Means and s.e.m. are shown (*n*=5).

References

1. Stover, C. K. et al. Complete genome sequence of *Pseudomonas aeruginosa* PAO1, an opportunistic pathogen. *Nature* **406**, 959-964 (2000).
2. Wang, M. et al. Quorum sensing and policing of *Pseudomonas aeruginosa* social cheaters. *Proc. Natl. Acad. Sci. U.S.A.* **112**, 2187-2191 (2015).
3. Rietsch, A. et al. ExsE, a secreted regulator of type III secretion genes in *Pseudomonas aeruginosa*. *Proc. Natl. Acad. Sci. U.S.A.* **102**, 8006-8011(2005).
4. Choi, K. H. et al. mini-Tn7 insertion in bacteria with secondary, non-*glmS*-linked attTn7 sites: example *Proteus mirabilis* HI4320. *Nat. Protoc.* **1**, 170-178 (2006).
